# Newborn First Feed and Prelacteal Feeds in Mansoura, Egypt

**DOI:** 10.1155/2014/258470

**Published:** 2014-05-06

**Authors:** Abdel-Hady El-Gilany, Doaa M. Abdel-Hady

**Affiliations:** Faculty of Medicine, Mansoura University, Mansoura 35516, Egypt

## Abstract

*Background*. Prelacteal feed (feeding any other substance before first breastfeeding) appears to be common despite its harmful effects. By definition a child provided with prelacteal feed (PLF) is not exclusively breastfed and PLF has many implications for the success and early initiation of breastfeeding. *Objectives*. To describe the prevalence of, nature of, and reasons for and factors associated with PLF. *Methods*. 647 mother-infant dyads were studied. Data was collected about the sociodemographic features of the family and baby, maternity care, the type of first feed before suckling, and causes of PLF. Maternal weight and height were measured and body mass index was calculated. *Results*. About 58% of newborns received prelacteal feeds. The commonest PLF was sugar/glucose water (39.6%). The most frequent reasons for giving PLF are tradition (61.0%) and mother's/mother in law's advice (58.3%). The logistic regression revealed that the independent predictors of PLF are urban residence; maternal education; father's education; low, middle, and high social class; maternal obesity; receiving antenatal care at private clinics and no antenatal care; Caesarean section; female babies; low birth weight; and admission to neonatal intensive care. *Conclusion*. Indiscriminate use of PLF should be discouraged in medical education and in antenatal maternal health education.

## 1. Introduction

The feeding of newborns has implications for immediate and future health. Colostrum is highly nutritious and immunogenic [1, 2]. However, its avoidance has been reported across the globe and prelacteal foods (PLFs) are introduced when breastfeeding is delayed [3]. PLFs are these foods given to newborn before breastfeeding is established or before breast milk “comes in,” usually on the first day of life [4, 5]. WHO and UNICEF summarized the ten steps to successful breastfeeding; these steps include prohibiting prelacteal feeding [6, 7].

Newborns are given PLF for different reasons including the following: to clean baby's bowels, keep mouth and throat moist, keep baby warm, soothe the baby, relief pain, and allow stool to be passed [8, 9]. Some in the Muslim community use PLF in the first day following birth because of the belief that colostrum has little nutritional value, may be considered dirty, and can even be harmful [8]. They may give sugar or water to the newborn instead of colostrum [10].

PLF may lead to lactation failure, insufficient milk production, infection, diarrhea, and short duration of breastfeeding [11–14]. It is noticed that there is a vicious cycle between PLF and delayed breastfeeding initiation; thus, PLF may delay the production of breast milk and the perceived lack of breast milk may encourage the use of PLF [15]. For these reasons WHO/UNICEF discourages the use of PLF unless medically indicated [16].

Knowing the determinants of introduction of PLF is essential to promote exclusive breastfeeding and early initiation of breastfeeding [17, 18]. There are many studies on PLF during the first three days after birth and it is not clear whether these are first feed or not. To the best of the authors' knowledge no studies were carried out in Egypt to describe the type of first feed given to the newborns. The objectives of this study are to describe the nature of the first feed as well as prevalence of, reasons for, and factors associated with prelacteal feeds (PLFs) in Mansoura, Egypt.

## 2. Material and Methods

This is a cross-sectional study done in Mansoura District during a period of 6 months (from July 1 to the end of December 2013). The target population is singleton newborns and their mothers. Mothers were interviewed during birth registration at the chosen health facilities. Birth registration usually takes place within the first week of birth.

Sample size was calculated online (https://www.dssresearch.com/KnowledgeCenter/toolkitca culators/samplesizecalculators.aspx). A pilot study on 50 newborns revealed that 50% received PLF other than colostrum in the first feed after birth. With a 5% precision, 5% alpha error, and 20% beta error the expected sample size should be 616 infants at least.

In the urban area 6 out of 11 health offices were selected. In the rural areas 19 out of 38 rural health units/family health units were selected. These were selected by systematic random sample from the lists of health offices and rural health units/family health units. The sample was distributed proportionally according to the number of registered births in each of the chosen facilities during the previous year. Trained nurses interviewed mothers during the birth registration and completed the questionnaire.

The questionnaire covered the sociodemographic data of the mother and her family, antenatal care, and place and mode of delivery. Infant's birth order, sex, birth weight, and gestational age were recorded. Mother's weight and height were measured according to standard precautions. Height was measured with a stadiometer accurate to 0.1 cm, with mother standing without shoes. Body weight was measured with calibrated electronic Seca scale (Seca Ltd., Birmingham, UK) accurate to 0.1 Kg, with subject wearing the lightest possible clothes. The BMI was calculated using the formula weight (in Kg)/squared height (in meters). BMI values were classified into two groups: normal weight and overweight/obese [19].

Social score of the family was calculated according to El-Gilany et al. (2012) [20]. This score encompasses parental education and work, family income, crowing index, household appliances and equipment, and usual source of health care. The total score was categorized into four levels of social classes.

Mothers were asked about the type of food/drink given to baby for the first time. If PLF other than colostrum was given, mothers were asked about the reasons for this practice.

The outcome variable is the prelacteal feed. It is defined as any food/liquid other than breast milk given to the infant before initiating breastfeeding for the first time [21, 22].

### 2.1. Ethical Consideration

The study was approved by both the ethical committee of College of Medicine, Mansoura University, and the local Health Directorate. Mothers gave informed verbal consent to participate in the study, before the interview.

### 2.2. Data Analysis

Data was analyzed using SPSS version 16. Variables were described as number and percent. In categorical variables *χ* ^2^ test and unadjusted odds ratio (OR) were used for comparison between groups. Significant predictors of prelacteal feed in bivariate analysis were entered into a logistic regression using the forward Wald methods and adjusted odds ratio (AOR) was calculated. *P* ≤ 0.05 was considered statistically significant.

## 3. Results

Table 1 shows that 42.2% and 57.8% of newborns received colostrum and prelacteal feeds in the first feed, respectively. The commonest PLFs were sugar/glucose water (39.6%), infant formula (28.6%), and herbs/decoction (21.7%). The most frequent reasons for giving PLF are tradition (61.0%), mother's/mother in law's advice (58.3%), keeping mouth and throat moist (55.9%), lack of/delay in milk production (47.9%), and advice of health care provider (42.0%).

Table 2 shows that PLF is significantly more reported in urban than rural areas (OR = 3.5), with highly educated mothers (OR = 2.0 for secondary education and OR = 1.9 for higher education), in low, middle and high social classes (OR = 1.7, 2.2 and 4.6; respectively) and on obese/overweight mothers (OR = 2.0).

PLF is significantly more encountered among women who received antenatal care at private clinics (OR = 2.0) and those who never received antenatal care (OR = 4.1), with delivery in private clinic/hospitals (OR = 3.4), with Caesarean section (OR = 3.1), female infants (OR = 1.8), low birth weight and preterm (OR = 4.1 and 1.9, resp.), and among infants admitted to ICU (OR = 3.8) (Table 3).

The logistic regression revealed that the independent predictors of PLF are urban residence (AOR = 3.8); maternal education (AOR = 0.6 and 1.5 for secondary and higher education, resp.); father's education (AOR = 3.0); low, middle, and high social class (AOR = 5.7, 24.3, and 33.8, resp.); maternal obesity (ARO = 1.7); receiving antenatal care at private clinics and no antenatal care (AOR = 11.7 and 3.8, resp.); Caesarean section (AOR = 2.1); female babies (AOR = 1.7); low birth weight (AOR = 4.2); and admission to neonatal intensive care (AOR = 3.5) (Table 4).

## 4. Discussion

The best practice in infant feeding is to put the infant at the breast as soon as practicable after delivery and to offer colostrum to the infant.

The practice of PLF is still common in Mansoura, Egypt. This study revealed that 57.8% of newborns were given different types of PLF as their first feed. This rate is intermediate among reported rates from previous studies. In Kuwait, PLF is the norm as 81.8% of infants receive PLF as their first feed [23]. In China 26% of hospital births were given formula, water, or milk as their first feed [24]. Most of the previous studies deal with PLF during the first three days after birth, irrespective of the nature of the first feed. Previous studies from Egypt reported PLF rates of 60% in a rural area [11]; 56% in Mansoura, the locality of the current study [25]; and 47% at the national level [26]. More or less similar rates were reported from Nigeria (56%) [27], Philippines (55%) [28], and Ethiopia (63%) [3]. Lower rates of PLF were reported from Libya (18.5%) [29], Uganda (31.3%) [30], Nepal (26.5%) [31], Kenya (26.8%) [32], Thailand (34.6%) [33], and India (33.3% up to 43.2) [14, 34–37].

A much lower rate was reported from Malawi (5.4%) [38] and much higher rates (96%) were reported from India [39–41] and Bangladesh (more than 92%) [42].

The independent predictors of PLF were urban residence; maternal education; father's education; low, middle, and high social class; maternal obesity; receiving antenatal care at private clinics and no antenatal care; Caesarean section; female babies; low birth weight; and admission to neonatal intensive care. One or more of these predictors were reported from previous studies in different countries. The Egypt Demographic and Health Survey found that PLF was more reported in medically assessed delivery, delivery at private health facilities, but no variation was noticed with infant sex, residence, mother's education and work status, or wealth quintiles [26]. In Uganda and Nepal regression analysis revealed that PLF was more with attending antenatal care, urban residence, Caesarean delivery, nonworking and noneducated mothers, less number of antenatal care visits, and first born babies [30, 31]. In China the independent predictors of PLF are NICU admission (AOR = 17.8) and high maternal education (AOR = 0.6) [24].

In different Indian studies PLF was more frequent in female babies and less educated mothers [14] and more in high income groups [41], in home births, and in illiterate mothers [36, 37]. In Malawi high PLF was reported in rural children and births outside health facilities [38]. In Nigeria PLF decreased with higher maternal education and high wealth [27]. In Philippines, PLF was more frequent in children of wealthier families and better educated mothers and children whose mothers were assisted by health professional during delivery [28]. These variations in the predictors of PLF necessitate each country to develop it own target population for intervention activities.

The commonest PLFs were sugar/glucose water, infant formula, and herbs/decoction. This agrees with previous studies in Egypt where glucose, herbal drinks [2, 43] sugar water and teas [11] were the most frequently used prelacteal feeds. In many African countries including Libya [29], Kenya [32], Nigeria [27], and Nepal [31], sugar water, glucose, plain water, and infant formula were the commonest PLFs. These PLFs were also reported in Philippines [28]. However, in India and Bangladesh the common feeds were honey, herbs, sugar water, gripe water, and cow's milk [34, 35, 37, 40–42]. This variation in the type of PLF between different countries could be attributed to the difference in culture, local beliefs, and availability of different feeds.

The most frequent reasons for giving PLF are tradition (61.0%), mother's/mother in law's advice (58.3%), keeping mouth and throat moist (55.9%), and lack of/delay in milk production (47.9%). This reveals the role of traditions and the influence of relatives in widespread practice of PLF. It is important to notice that advice of health care provider is cited as a reason by 42% of mothers. This highlights the importance of medical and paramedical education and the continuation of in-service training in breastfeeding practice. Previous studies in Egypt found that lack of milk in mothers' breast (74%), maternal exhaustion or illness following labor (29%) [11], and breastfeeding difficulties (engorgement, flat nipple, sore, and inflammation) were the commonest causes of PLF [26, 43]. A previous study in Nigeria found that about 70% and 27% of doctors and nurses prescribe PLF routinely and in special circumstances, respectively. Their reasons were perceived milk insufficiency, prevention of dehydration, hypoglycemia, and neonatal jaundice, and well as cleansing the baby's gut and rest the mother [5]. Indian studies reported that PLFs were given to clean infants systems [40], being traditional belief as they considered colostrum thick, cheesy, indigestible, unhygienic, and not good for the baby [35]. In Bangladesh, tradition, child becoming normal and quiet, delayed milk suction, and clearing newborn's oral cavity were the most cited reasons for giving PLFs [42]. All these reasons are amenable for prevention through appropriate education.

One of the strengths of this study is low possibility of recall bias as data was collected within few days after birth. There are several limitations that should be considered when interpreting the results of this study. We did not collect data about PLF during the first three days of life as most of the newborns were registered before this duration. Also the sample was restricted to newborns in Mansoura District and a large scale nationwide study is needed to document the practices in other regions of Egypt.

## 5. Conclusions

Although the Egyptian authorities have set breastfeeding policies consistent with international recommendations, many neonates are given PLF. PLF is still a factor to be targeted through educational intervention. Further education of the mothers and health staff about adverse effects of PLF is required. It is important to emphasize the nutritional value of colostrum and misconceptions about PLFs through a culturally acceptable approach.

## Figures and Tables

**Table 1:** Type of first feed, PLFs, and their reasons.

	Number	% of total	% of PLF
Type of first feed			
Colostrum	273	42.2	
PLF	374	57.8	
Type of PLF			
Sugar or glucose water	148	22.9	39.6
Infant formula	107	16.5	28.6
Herbs/decoction*	81	12.5	21.7
Animal milk diluted with water	19	2.9	5.1
Plain water	7	1.1	1.9
Gripe water**	5	0.8	1.3
Tea	3	0.5	0.8
Soft drinks	2	0.3	0.5
Juices (fresh or canned)	2	0.3	0.5
Reasons for PLFs^@^			
Tradition/convention	228	35.2	61.0
Mother's/mother in law's advice	218	33.7	58.3
Keeping mouth and throat moist	209	32.3	55.9
Lack of/delay in milk production	179	27.7	47.9
Advice of health care provider	157	24.3	42.0
Infant refused suckling	144	22.3	38.5
Maternal exhaustion/illness	112	17.3	29.9
To clean infant's gut/throat/mouth	105	16.2	28.1
Breast problems (e.g., mastitis,engorgement, and soreness)	102	15.8	27.3
To calm/soothe the baby	94	14.5	25.1
Colostrum is bad to baby	92	14.2	24.6
Allowing stool to be passed	89	13.8	23.8
Infant sickness/ICU admission	86	13.3	23.0
Nurture baby	73	11.3	19.5
Keeping baby warm	16	2.5	4.3

*Such as cumin, caraway, cinnamon, aniseed, and chamomile.

**Commercial preparation for soothing colicky babies.

^@^Categories are not mutually exclusive.

ICU: intensive care unit.

**Table 2:** Prelacteal feeding before first suckling according to maternal characteristics.

	Total	Prelacteal feeding *N* (%)	*P* value	OR (95% CI)
Overall	647	374 (57.8)		(54.0–61.6)
Residence				
Rural	393	183 (46.6)		1 (r)
Urban	254	191 (75.2)	≤0.001	3.5 (2.4–5.0)
Mother's age				
<20 years	279	171 (62.4)		1 (r)
20–<35 years	319	172 (53.9)	0.04	0.7 (0.5–0.99)
35 and more	54	31 (57.4)	0.5	08 (0.4–1.5)
Mother's education				
<secondary	104	57 (54.8)		1 (r)
Secondary	316	165 (52.2)	0.6	0.9 (0.6–1.4)
>secondary	227	152 (67.0)	0.003	1.7 (1.01–2.8)
Mother's work				
No	502	293 (58.4)		1 (r)
Yes	145	81 (55.9)	0.6	0.9 (0.6–1.3)
Father's education				
<secondary	122	54 (44.3)		1 (r)
Secondary	295	182 (61.7)	0.001	2.0 (1.3–3.2)
>secondary	230	138 (60.0)	0.005	1.9 (1.2–3.0)
Father's work				
Farmer/manual worker	326	181 (55.5)		1 (r)
Professional/employee	192	119 (62.0)	0.2	1.3 (0.9–1.9)
Trades/business/others	129	74 (57.4)	0.7	1.1 (0.7–1.7)
Family income				
Sufficient	366	220 (60.1)		1 (r)
Insufficient	281	154 (54.8)	0.2	0.8 (0.6–1.1)
Social class				
Very low	226	98 (43.4)		1 (r)
Low	127	71 (55.9)	0.02	1.7 (0.04–2.6)
Middle	155	97 (62.6)	≤0.001	2.2 (1.4–3.4)
High	139	108 (77.7)	≤0.001	4.6 (2.8–7.6)
Maternal obesity				
No	267	128 (47.9)		1 (r)
Obese/overweight	380	246 (64.7)	≤0.001	2.0 (1.4–2.8)

OR: odds ratio; CI: confidence interval; r: reference group.

**Table 3:** Prelacteal feeding before first suckling according to antenatal care, delivery, and infant's characteristics.

	Total	Prelacteal feeding *N* (%)	*P* value	OR (95% CI)
Source of antenatal care				
Primary health care	100	46 (46.0)		1 (r)
Governmental hospital	76	34 (44.7)	0.9	0.95 (0.5–1.8)
Private clinics	439	275 (62.6)	0.002	2.0 (1.2–3.1)
>one source	14	5 (35.7)	0.5	0.7 (0.2–2.3)
None	18	14 (77.8)	0.01	4.1 (1.2–16.0)
Number of antenatal visits				
<5	158	86 (54.4)		1 (r)
5–9	328	179 (54.6)	0.98	1.01 (0.7–1.5)
10 and more	143	95 (66.4)	0.03	1.7 (1.01–2.7)
None	18	14 (77.8)	0.06	2.9 (0.9–11.1)
Place of delivery				
Home	60	22 (36.7)		1 (r)
Governmental hospital	191	89 (46.6)	0.2	1.5 (0.8–2.9)
Private clinic/hospital	396	263 (66.4)	≤0.001	3.4 (1.9–6.3)
Model of delivery				
Vaginal delivery	440	218 (49.5)		1 (r)
Caesarean section	207	156 (75.4)	≤0.001	3.1 (2.1–4.6)
Infant sex				
Male	348	179 (51.4)		1 (r)
Female	299	195 (65.2)	≤0.001	1.8 (1.3–2.5)
Birth order				
First born	244	154 (63.1)		1 (r)
2nd and 3rd	333	186 (55.9)	0.08	0.7 (0.5–1.1)
4th and more	70	34 (48.6)	0.03	0.6 (0.3–0.98)
Low birth weight				
No	598	333 (55.7)		1 (r)
Yes	49	41 (83.7)	≤0.001	4.1 (1.8–9.6)
Preterm				
No	556	310 (55.8)		1 (r)
Yes	91	64 (70.3)	0.009	1.9 (1.1–3.1)
NICU admission				
No	575	315 (54.8)		1 (r)
Yes	72	59 (81.9)	≤0.001	3.8 (1.9–7.4)

OR: odds ratio; CI: confidence interval; r: reference group; NICU: neonatal intensive care unit.

**Table 4:** Logistic regression analysis of the independent significant predictors of PLF before first suckling.

	*β*	*P*	AOR (95% CI)
Residence			
Rural	—		1 (r)
Urban	1.3	≤0.001	3.8 (2.4–6.0)
Mother's education			
<secondary	—		1 (r)
Secondary	−0.9	0.5	0.6 (0.2–1.2)
>secondary	1.3	≤0.001	1.5 (1.1–2.3)
Father's education			
<secondary	—		1 (r)
Secondary	1.1	≤0.001	3.0 (1.7–5.3)
>secondary	0.2	0.6	1.2 (0.6–2.6)
Social class			
Very low	—		1 (r)
Low	1.7	≤0.001	5.7 (2.9–11.4)
Middle	3.5	≤0.001	24.3 (12.0–37.9)
High	3.8	≤0.001	33.8 (24.1–39.8)
Maternal obesity			
No	—		1 (r)
Obese/overweight	0.5	0.05	1.7 (1.1–2.8)
Source of antenatal care			
Primary health care	—		1 (r)
Governmental hospital	−0.2	0.7	0.8 (0.4–1.9)
Private clinics	2.5	≤0.001	11.7 (2.2–19.4)
>one source	1.7	0.08	1.4 (0.6–5.4)
None	1.3	0.04	3.8 (1.9–5.2)
Model of delivery			
Vaginal delivery	—		1 (r)
Caesarean section	0.7	≤0.001	2.1 (1.2–3.2)
Infant sex			
Male	—		1 (r)
Female	0.5	0.01	1.7 (1.1–3.2)
Low birth weight			
No	—		1 (r)
Yes	1.4	0.004	4.2 (1.6–11.2)
NICU admission			
No	—		1 (r)
Yes	1.3	0.002	3.5 (1.6–7.8)
Constant	−1.3		
Model *χ* ^2^	262.9, *P* ≤ 0.001		
Percent correctly predicted	76.5		

AOR: adjusted odds ratio; CI: confidence interval; r: reference group.

## References

[1] Labbok MH, Clark D, Goldman AS (2004). Breastfeeding: maintaining an irreplaceable immunological resource. *Nature Reviews Immunology*.

[2] Kent JC (2007). How breastfeeding works. *Journal of Midwifery and Women’s Health*.

[3] Rogers NL, Abdi J, Moore D (2011). Colostrum avoidance, prelacteal feeding and late breast-feeding initiation in rural Northern Ethiopia. *Public Health Nutrition*.

[4] Laroia N, Sharma D (2006). The religious and cultural bases for breastfeeding practices among the Hindus. *Breastfeeding Medicine*.

[5] Akuse RM, Obinya EA (2002). Why healthcare workers give prelacteal feeds. *European Journal of Clinical Nutrition*.

[6] Vallenas C, Savage F (1998). *Evidenc for the Ten Steps to Successful Breastfeeding*.

[7] WHO/UNICEF: Baby-Friendly Hospital Initiative (2009). *Revised and Expanded for Integrated Care*.

[8] Fikree FF, Ali TS, Durocher JM, Rahbar MH (2005). Newborn care practices in low socioeconomic settlements of Karachi, Pakistan. *Social Science and Medicine*.

[9] Ahmed FU, Rahman ME, Alam MS (1996). Prelacteal feeding: influencing factors and relation to establishment of lactation. *Bangladesh Medical Research Council Bulletin*.

[10] Gatrad AR, Sheikh A (2001). Muslim birth customs. *Archives of Disease in Childhood: Fetal and Neonatal Edition*.

[11] Moshaddeque Hossain M, Radwan MM, Arafa SA, Habib M, DuPont HL (1992). Prelacteal infant feeding practices in rural Egypt. *Journal of Tropical Pediatrics*.

[12] Hossain MM, Reves RR, Radwan MM, Habib M, DuPont HL (1995). The timing of breastfeeding initiation and its correlates in a cohort of rural Egyptian infants. *Journal of Tropical Pediatrics*.

[13] WHO (1997). *Hypoglycemia of the Newborn. Review of the Literature. WHO/CHD/97.1, WHO/MSM/97.1*.

[14] Jagzape T, Lohkare A, Vagha J, Lakhkar BB Prevalence of prelacteal feeding practice in Wardha and the effect of antenatal education on it.

[15] Athavale AV, Athavale SA, Deshpande SG, Zodpey SP, Sangole S (2004). Initiation of breast-feeding by urban women. *Health and Population: Perspectives and Issues*.

[16] WHO/UNICEF (1990). *Innocent Declaration on the Protection, Promotion and Support of Breastfeeding. Breastfeeding in the 1990s. A Global Initiative*.

[17] Ulak M, Chandyo RK, Mellander L, Shrestha PS, Strand TA (2012). Infant feeding practices in Bhaktapur, Nepal: a cross-sectional, health facility based survey. *International Breastfeeding Journal*.

[18] Chandrashekhar TS, Joshi HS, Binu VS, Shankar PR, Rana MS, Ramachandran U (2007). Breast-feeding initiation and determinants of exclusive breast-feeding—a questionnaire survey in an urban population of western Nepal. *Public Health Nutrition*.

[19] WHO (1995). Physical status, “the use and interpretation of anthropometry”, Report of a WHO Expert committee. *WHO Technical Report*.

[20] El-Gilany A, El-Wehady A, El-Wasify M (2012). Updating and validation of the socioeconomic status scale for health research in Egypt. *Eastern Mediterranean Health Journal*.

[21] Galal O (1995). Child feeding patterns in the Middle East. *Saudi Journal of Gastroenterology*.

[22] Ludvigsson JF (2003). Breastfeeding intentions, patterns, and determinants in infants visiting hospitals in La Paz, Bolivia. *BMC Pediatrics*.

[23] Dashti M, Scott JA, Edwards CA, Al-Sughayer M (2010). Determinants of breastfeeding initiation among mothers in Kuwait. *International Breastfeeding Journal*.

[24] Qiu L L, Xie X, Lee A, Binns C (2007). Infant's first feeds in Hangzlou, PR China. *Asia Pacific Journal of Clinical Nutrition*.

[25] EL-Gilany A, Badawy K (2013). Breastfeeding performance index at age of 6 months in Mansoura, Egypt. *TAF Preventive Medicine Bulletin*.

[26] El-Zanaty F, Way A (2009). Feeding practices and micronutrient supplementation. *Egypt Demographic and Health Survey 2008*.

[27] (2009). *Nigeria Demographic and Health Survey 2008*.

[28] (2009). *Philippines National Demographic and Health Survey 2008*.

[29] Shembesh NM, Balo NNM, Singh R (1997). Breast-feeding and weaning patterns in Benghazi, Libyan Arab Jamahiriya. *Eastern Mediterranean Health Journal*.

[30] Ogah AO, Ajayi AM, Akin S, Okolo SN (2012). A cross-sectional study of prelacteal feeding practice among women attending Kampala International University Teaching Hospital Maternal and Child Health Clinic, Bushenyi, Western Uganda. *Asian Journal of Medical Sciences*.

[31] Khanal V, Adhikari M, Sauer K, Zhao Y (2013). Factors associated with the introduction of prelacteal feeds in Nepal: findings from the Nepal Demographic and Health Survey 2011. *International Breastfeeding Journal*.

[32] Lakati AS, Makokha OA, Binns CW, Kombe Y (2010). The effect of pre-lacteal feeding on full breastfeeding in Nairobi, Kenya. *East African Journal of Public Health*.

[33] Senarath U, Dibley MJ, Agho KE (2007). Breast-feeding performance index: a composite index to describe overall breast-feeding performance among infants under 6 months of age. *Public Health Nutrition*.

[34] Spana P, Ameya H, Rooma P, Aarti P, Rashed AK, Narayan KA (2009). Prevalence of exclusive breastfeeding and its correlates in an urban slum in western India. *International e-Journal of Science, Medicine & Education*.

[35] Dakshayani B, Gangadhar MR (2008). Breast feeding practices among the Hakkipikkis: a tribal population of Mysore District, Karnataka. *Ethno Medicine*.

[36] Kulkarni RN, Anjenaya S, Gujar R (2004). Breastfeeding practices in an urban community of Kalamboli, Navi Mumbai. *Indian Journal of Community Medicine*.

[37] Joseph N, Unnikrishnan B, Naik VA (2013). Infant rearing practices in South India: a longitudinal study. *Journal of Family Medicine and Primary Care*.

[38] (2005). *Malawi Demographic and Health Survey 2004*.

[39] Prasad B, De Costello LAM (1995). Impact and sustainability of a “baby friendly” health education intervention at a district hospital in Bihar, India. *British Medical Journal*.

[40] Bandyopadhyay M (2009). Impact of ritual pollution on lactation and breastfeeding practices in rural West Bengal, India. *International Breastfeeding Journal*.

[41] Rania SK, Mengi V, Singh G (2012). Determinants of prelacteal feeding among infants of RS Pura block of Jammu and Kashmir, India. *Journal of Family Medicine and Primary Care*.

[42] Tarannum S, Z Hyder SM (1998). Pre-lacteal feeding practices in a rural area of Bangladesh. *Working Paper*.

[43] Al Ghwass MME, Ahmed D (2011). Prevalence and predictors of 6-month exclusive breastfeeding in a rural area in Egypt. *Breastfeeding Medicine*.

